# Lipopolysaccharide (LPS) stimulation of fungal secondary metabolism

**DOI:** 10.1080/21501203.2014.930530

**Published:** 2014-07-22

**Authors:** Zeinab G. Khalil, Pabasara Kalansuriya, Robert J. Capon

**Affiliations:** ^a^ Institute for Molecular Bioscience, The University of Queensland, St Lucia, QLD4072, Australia

**Keywords:** lipopolysaccharide, stimulation, activation, enhancement, acceleration, silent secondary metabolism

## Abstract

We report on a preliminary investigation of the use the Gram-negative bacterial cell wall constituent lipopolysaccharide (LPS) as a natural chemical cue to stimulate and alter the expression of fungal secondary metabolism. Integrated high-throughput micro-cultivation and micro-analysis methods determined that 6 of 40 (15%) of fungi tested responded to an optimal exposure to LPS (0.6 ng/mL) by activating, enhancing or accelerating secondary metabolite production. To explore the possible mechanisms behind this effect, we employed light and fluorescent microscopy in conjunction with a nitric oxide (NO)-sensitive fluorescent dye and an NO scavenger to provide evidence that LPS stimulation of fungal secondary metabolism coincided with LPS activation of NO. Several case studies demonstrated that LPS stimulation can be scaled from single microplate well (1.5 mL) to preparative (>400 mL) scale cultures. For example, LPS treatment of *Penicillium* sp. (ACM-4616) enhanced pseurotin A and activated pseurotin A_1_ and pseurotin A_2_ biosynthesis, whereas LPS treatment of *Aspergillus* sp. (CMB-M81F) substantially accelerated and enhanced the biosynthesis of shornephine A and a series of biosynthetically related ardeemins and activated production of neoasterriquinone. As an indication of broader potential, we provide evidence that cultures of *Penicillium* sp. (CMB-TF0411), *Aspergillus niger* (ACM-4993F), *Rhizopus oryzae* (ACM-165F) and *Thanatephorus cucumeris* (ACM-194F) were responsive to LPS stimulation, the latter two examples being particular noteworthy as neither are known to produce secondary metabolites. Our results encourage the view that LPS stimulation can be used as a valuable tool to expand the molecular discovery potential of fungal strains that either have been exhaustively studied by or are unresponsive to traditional culture methodology.

## 1. Introduction

Historically, the pharmaceutical industry has relied heavily on microbial natural products, which represent an extraordinarily diverse, preassembled pool of biologically active molecules, programmed by evolution to be potent and selective modulators of key biopolymers, cells, tissues, organs and living systems (plants, animals and microbes). Knowledge of nature’s biosynthetic equivalent to ‘intellectual property’ reveals privileged structures that can inform and inspire modern drug discovery, repurposing ecological advantage to pharmaceutical benefit. Fungi, for example, have been the source of some of the most therapeutically and commercially successful drugs in history, including the antibiotic penicillins and the antilipidemic statins. The journey to discover, develop, manufacture and market microbe-inspired drugs has sparked and fuelled a revolution in global science, business and health care. Notwithstanding historic successes, by late last century the pharmaceutical industry had distanced itself from microbe-inspired drug discovery, citing inefficiencies imposed by excessive rediscovery of known microbial metabolites. This retreat from microbe-inspired drug discovery heralded a period of record high R&D expenditure, delivering record numbers of new chemical entities, which equate to new drugs, and culminating in a seriously depleted drug discovery pipeline, underscoring an emerging threat to future health care. Confronted by rapidly emerging multi-drug resistance, and the need to deliver safer, cheaper and better drugs on a global scale, for a wider array of diseases and indications, the time is right to revisit and re-engage with microbe-inspired drug discovery. Supportive of such a reinvestment, the last three decades have witnessed remarkable advances across the fields of microbiology, genetics, biology and chemistry, disclosing a wealth of secondary metabolite genes ‘running silent’ under traditional culture strategies. This report describes a preliminary investigation of the use of the Gram-negative bacterial cell wall constituent lipopolysaccharide (LPS) as a natural chemical cue to stimulate and alter the transcriptional status of underperforming and/or silent secondary metabolism. As LPS is known to activate inducible nitric oxide synthase (iNOS)-mediated inflammatory responses in mammalian cells, we hypothesize that LPS induces a comparable biochemical response in fungal cells, stimulating the production of ‘defensive’ secondary metabolites. To test this hypothesis, we implemented integrated high-throughput (HTP) micro-bioreactor cultivation and micro-analysis methods to probe the effect of LPS on a set of 40 fungi. By leveraging advantages of HTP cultivation, we detected multiple occurrences where LPS-treated fungal cultures exhibited significant changes in secondary metabolite profiles. Herein, we provide an analysis of several such occurrences.

## 2. Materials and methods

### 2.1. Materials

A sample of LPS isolated from *Escherichia coli* (0111:B4) was purchased from Sigma-Aldrich (Sydney, NSW, Australia). Yeast extract was purchased from Merck (Darmstadt, Germany); peptone from Oxoid (Basingstoke, Hampshire, UK); malt extract, starch and agar from Sigma-Aldrich; glucose from Biochemicals (Sydney, NSW, Australia) and Sabouraud dextrose broth (SDB) medium and Tryptic soy agar/broth were from BD Diagnostics (Burlington, NC, USA). All solvents were analytical or high-pressure liquid chromatography (HPLC) grade and filtered/degassed through 0.45 μM polytetrafluoroethylene membrane prior to use. Deuterated solvents were purchased from Cambridge Isotopes (Tewksbury, MA, USA). International Streptomyces Project (ISP-2) (Ayari et al. 2012) and M1 (Raju et al. 2010) media were prepared as previously reported. The NO detection kit (ENZ-51013) including the NO scavenger 2-(4-carboxyphenyl)-4,4,5,5-tetramethylimidazoline-1-oxyl-3-oxide (c-PTIO) and matching 10× wash buffer was purchased from Enzo Life Sciences (Sydney, NSW, Australia).

### 2.2. Instrumentation

Applikon micro-Flasks (micro-bioreactors), also known as the System Duetz (www.enzyscreen.com), were purchased from Enztech Pty Ltd (Sydney, NSW, Australia). All strains were handled in a Laftech class II biological safety cabinet and were cultivated in either an MMM Friocell 111 incubator from Lomb Scientific or an Innova 42R incubator shaker (John Morris, Sydney, NSW, Australia) or a Contherm CON1100 incubator (Contherm Scientific Ltd, Korokoro, Lower Hutt, New Zealand) and supplemented with MaxQ 6000 shaker (ThermoFisher, Melbourne, VIC, Australia). Optical density (OD_600nm_) measurements and UV-visible spectra were acquired on a Cary 50 spectrophotometer in 1 cm quartz or polypropylene cells, respectively. Analytical ultra-high-pressure liquid chromatography (UPLC-DAD) was performed on an Agilent 1290 Infinity system equipped with a diode array detector. Analytical high-pressure liquid chromatography mass spectrometry (HPLC-DAD-ESIMS) was performed on an Agilent 1100 Series separation module equipped with a diode array and an Agilent 1100 single quadrupole mass detector, the latter operating in dual positive and negative ion modes. Semi-preparative and preparative HPLC was performed on an Agilent 1100 series separation module equipped with an auto-sampler, diode array detector and fraction collector. Nuclear magnetic resonance spectra obtained on a 600 MHz Bruker Avance III spectrometer with a TCI ^1^H/^13^C/^15^N/Z-GRD cryoprobe and were referenced to residual ^1^H and ^13^C signals in the deuterated solvents. High-resolution mass spectrometry measurements were obtained on a Bruker micrOTOF mass spectrometer by direct infusion in acetonitrile (MeCN) at 3 μL/min using sodium formate clusters as an internal calibrant. Chiroptical measurements ([*α*]_D_) were obtained on a JASCO P-1010 polarimeter in a 100 × 2 mm cell. Confocal microscopy was performed on a Zeiss LSM510 confocal laser-scanning microscope equipped with a Zeiss 63x/1.4NA oil-immersion objective. Confocal microscopy samples, in 12 mm coverglass bottom culture dishes (ProSciTech), were excited with a 543 nm He–Ne laser and measured emissions filtered through a 560 nm long-pass filter.

### 2.3. Fungal strains and culture conditions

Fungal strains (×40) were selected from our in-house microbe library comprising isolates from Australian terrestrial and marine substrata and commercial repositories. Selected isolates were cultivated on ISP-2 agar plates at 27°C for 15 days. To prepare inocula for activation trials, a 1 cm^2^ plug excised from each agar plate was incubated at 190 rpm at 27°C for 10-15 days in a shake flask (250 mL) containing ISP-2 or M1 liquid broth (80 mL). After incubation, an aliquot (1 mL) of the culture broth was diluted to OD_600 nm_ 0.01 (10^6^–10^8^ cfu/mL) to generate an inoculum that was cryo-preserved in 20% aqueous glycerol at −80°C. Seed cultures of *Penicillium* sp. (ACM-4616), *Penicillium* sp. (CMB-TF0411) and *Aspergillus* sp. (CMB-M81F) were prepared by growing inoculum (1 mL) in shake flasks (250 mL) containing SDB, ISP-2 or M1 3.3% ocean sea salt broth (80 mL), respectively, at 190 rpm and 26.5°C over 7 days. Large-scale cultures were prepared at 190 rpm and 26.5°C in multiple Fernbach flasks (2 L) charged with seed culture (5 mL) and SDB, ISP-2 or M1 3.3% ocean sea salt broth (395 mL) treated with LPS (26.6 μL, 0.6 ng/mL), over the time periods as specified in Sections 2.8.2, 2.9.2 and 2.10.2.

### 2.4. Micro-bioreactor cultures

Each well in the micro-bioreactor 24-well plate was loaded with culture media (1.5 mL) specific to each fungal strain (Sections 2.8–2.10), with 20 wells inoculated with fungal cells (15 μL), and four wells retained as negative controls (i.e. media only). Micro-bioreactor plates were incubated at 190 rpm at 27°C until optimal growth was achieved (OD_600 nm_ = 0.1, 10^10^−10^12^ cfu/mL). Typical culture times ranged from 5 to 7 days, although some cultures required longer incubation times (e.g. >120 days). Cultures were extracted *in situ* with EtOAc (ethyl acetate) (2 mL), after which the decanted organic layer was dried under N_2_ and resuspended in MeOH (methanol) (100 μL) to generate analytes that were analysed by UPLC-DAD to document baseline secondary metabolite production.

### 2.5. LPS stimulation of fungal secondary metabolism

An aqueous stock solution of LPS (1 mg/mL) was serially diluted and aliquots (15 μL) dispensed in duplicate across 20 wells of a (24-well) micro-bioreactor preloaded with fungal culture media (1470 μL) and inoculum (15 μL), with four uninoculated wells retained as negative controls. Micro-bioreactor plates were cultivated and extracted *in situ* to generate analytes as detailed in Section 2.4. Following this approach, we set up an LPS activation test matrix on 40 fungal strains at concentrations of 100, 33, 11, 3.6, 1.2, 0.4, 0.1, 0.04, 0.01 and 0 ng/mL. LPS-mediated stimulation events were monitored by UPLC-DAD analysis to determine whether the presence of LPS altered secondary metabolite profiles. Analytes indicative of LPS stimulation were also analysed by HPLC-DAD-ESIMS.

### 2.6. UPLC-DAD analysis

Aliquots (1 μL) from >1000 analytes (see Sections 2.4 and 2.5) were analysed by UPLC-DAD employing an Agilent Zorbax C_8_ RRHD 1.8 μm (2.1 × 50 mm) column, with a 2.50 min gradient elution at 0.417 mL/min from 90% H_2_O/MeCN (acetonitrile) to 100% MeCN and inclusive of an isocratic 0.01% trifluoroacetic acid (TFA) modifier, with online detection at 210 and 254 nm. All data were analysed using Agilent Open Lab CDS Rev.C.01.03 (37) ChemStation software. These analyses detected six LPS stimulation events associated with *Penicillium* sp. (ACM-4616), *Penicillium* sp. (CMB-TF0411), *Aspergillus* sp. (CMB-M81F), *Aspergillus niger* (ACM-4993F), *Rhizopus oryzae* (ACM-165F) and *Thanatephorus cucumeris* (ACM-194F).

### 2.7. HPLC-DAD-ESIMS analysis

Aliquots (10 μL) from analytes indicative of LPS-mediated stimulation (Section 2.6) were analysed by HPLC-DAD-ESI(±)MS employing an Agilent Zorbax SB-C_8_ 5 μm (150 × 4.6 mm) column, with a 15 min gradient elution at 1 mL/min from 90% H_2_O/MeCN to 100% MeCN, followed by a 5 min hold and inclusion of an isocratic 0.05% formic acid (HCO_2_H) modifier. All data were analysed using Agilent ChemStation Rev.9.03A and Purify version A.1.2 software.

### 2.8. Penicillium sp. (ACM-4616)

#### 2.8.1. Micro-bioreactor cultivation and LPS stimulation of *Penicillium* sp. (ACM-4616)

*Penicillium* sp. (ACM-4616) was sourced in 1974 from a mushroom collected in Australia. A micro-bioreactor plate loaded with SDB medium and inoculated with *Penicillium* sp. (ACM-4616) was cultivated in the absence and presence of LPS for 7 days, and the resulting analytes analysed by UPLC-DAD and HPLC-DAD-ESIMS.

#### 2.8.2. Preparative cultivation of LPS-stimulated *Penicillium* sp. (ACM-4616)

Large-scale culture of *Penicillium* sp. (ACM-4616) was achieved in 6 × 2 L Fernbach flasks over 7 days as described in Section 2.3, after which each flask was extracted with EtOAc (400 mL) and the combined organic phases concentrated *in vacuo* to yield a total extract (165.3 mg). The total extract was sequentially triturated (8 mL aliquots) and concentrated *in vacuo* to yield hexane (4 mg), CH_2_Cl_2_ (159.4 mg) and MeOH (1.9 mg) soluble fractions. The CH_2_Cl_2_ soluble fraction was subjected to preparative reversed-phase HPLC on a Phenomenex Luna C_18_ 10 μm (250 × 21 mm) column, with a 15 min gradient elution at 20 mL/min from 90% H_2_O/MeCN to 100% MeCN and inclusive of an isocratic 0.01% TFA modifier, to yield *cyclo*-(L-Phe-L-Pro) (**1**) (3.3 mg, 2.0%), *cyclo*-(L-Trp-L-Pro) (**2**) (0.9 mg, 0.5%), pseurotin A (**3**) (2.0 mg, 1.2%), pseurotin A_1_ (**4**) (1.2 mg, 0.7%) and pseurotin A_2_ (**5**) (0.9 mg, 0.5%). The structures of **1−5** were determined by detailed spectroscopic analysis, and comparisons to the literature data, and authentic standards (where available) and per cent yields were determined on a mass-to-mass basis against the weight of the total extract.

### 2.9. Penicillium sp. (CMB-TF0411)

#### 2.9.1. Micro-bioreactor cultivation and LPS stimulation of *Penicillium* sp. (CMB-TF0411)

*Penicillium* sp. (CMB-TF0411) was isolated in 2009 from a soil sample collected from North Stradbroke Island, Brisbane. A micro-bioreactor plate loaded with ISP-2 medium and inoculated with *Penicillium* sp. (CMB-TF0411) was cultivated in the absence and presence of LPS for 10 days, and the resulting analytes were analysed by UPLC-DAD and HPLC-DAD-ESIMS.

#### 2.9.2. Preparative cultivation of LPS-stimulated *Penicillium* sp. (CMB-TF0411)

Large-scale culture of *Penicillium* sp. (CMB-TF0411) was achieved in 2 × 2 L Fernbach flasks over 7 days as described in Section 2.3, after which each flask was extracted with EtOAc (400 mL) and the combined organic phases concentrated *in vacuo* to yield a total extract (83.2 mg). The total extract was sequentially triturated (8 mL aliquots) and concentrated *in vacuo* to yield hexane (8 mg), CH_2_Cl_2_ (10 mg) and MeOH (45.2 mg) soluble fractions. The components of the MeOH soluble fraction were separated by semi-preparative reversed-phase HPLC using an Agilent Zorbax C_8_ 5 μm (250 × 9.4 mm) column, with a 30 min gradient elution at 3 mL/min from 90% H_2_O/MeCN to 10% H_2_O/MeCN and inclusive of an isocratic 0.01% TFA modifier, to yield (+)-deoxyluteoskyrin (**6**) (0.5 mg, 1.1%) (−)-rugluosin (**7**) (0.8 mg, 1.7%) and (–)-skyrin (**8**) (1.4 mg, 3.1%). The structures of **6–8** were determined by detailed spectroscopic analysis, and comparisons to the literature data, and authentic standards (where available) and per cent yields were determined on a mass-to-mass basis against the weight of the total extract.

#### 2.9.3. Time course study on LPS stimulation of Penicillium sp. (CMB-TF0411)

A micro-bioreactor plate loaded with ISP-2 medium, inoculated with *Penicillium* sp. (ACM-4616) and treated with LPS (0.6 ng/mL), was cultivated for 30 days. Every 5 days, the culture broth in two wells was extracted *in situ* with EtOAc (2 mL) to generate analytes (Section 3.4) that were subsequently analysed by UPLC-DAD. The titres of (+)-deoxyluteoskyrin (**6**) and (−)-rugulosin (**7**) were established by comparison with calibration curves generated from authentic standards.

### 2.10. Aspergillus sp. (CMB-M81F)

#### 2.10.1. Micro-bioreactor cultivation and LPS stimulation of *Aspergillus* sp. (CMB-M81F)

*Aspergillus* sp. (CMB-M81F) was isolated in 2007 from a marine sediment sample obtained from Shorncliffe, Australia. A micro-bioreactor plate loaded with M1 medium and 3.3% artificial sea salt (Ocean Nature), and inoculated with *Aspergillus* sp. (CMB-M81F), was cultivated in the absence and presence of LPS for 120 days, and the resulting analytes were analysed by UPLC-DAD and HPLC-DAD-ESIMS.

#### 2.10.2. Preparative cultivation of LPS-stimulated *Aspergillus* sp. (CMB-M81F)

Large-scale culture of *Aspergillus* sp. (CMB-M81F) was achieved in 2 × 2 L Fernbach flasks over 120 days as described in Section 2.3, after which each flask was extracted with EtOAc (400 mL) and the combined organic phases concentrated *in vacuo* to yield a total extract (100.5 mg). The total extract was sequentially triturated (15 mL aliquots) and concentrated *in vacuo* to yield hexane (6.3 mg) and CH_2_Cl_2_ (85.6 mg) soluble fractions. The CH_2_Cl_2_ soluble fraction was subjected to semi-preparative reversed-phase HPLC using an Agilent-Zorbax C_8_ 5 μm (250 × 9.4 mm) column, with a 30 min gradient elution at 3 mL/min from 90% H_2_O/MeCN to 100% MeCN, to yield shornephine A (**9**) (2 mg, 2.3%), 15b-β-hydroxy-5-*N*-acetylardeemin (**10**) (1.5 mg, 1.7%), 5-*N*-acetylardeemin (**11**) (1.9 mg, 2.2%), 15b-β-methoxy-5-*N*-acetylardeemin (**12**) (1.5 mg, 1.7%) and neoasterriquinone (**13**) (1.1 mg, 1.2%). The structures of **9–13** were determined by detailed spectroscopic analysis, and comparisons to the literature data, and authentic standards (where available) and per cent yields were determined on a mass-to-mass basis against the weight of the total extract.

#### 2.10.3. Time course study on LPS stimulation of *Aspergillus* sp. (CMB-M81F)

A micro-bioreactor plate loaded with M1 broth and 3.3% artificial sea salt, inoculated with *Aspergillus* sp. (CMB-M81F) and treated with LPS (0.6 ng/mL), was cultivated for 160 days. Every 5 days, the culture broth in two wells was extracted *in situ* with EtOAc (2 mL) for UPLC-DAD analysis. Titres of shornephine A (**9**) and neoasterriquinone (**13**) were established by comparison with calibration curves generated from authentic standards.

### 2.11. Calibration curves

Duplicate aliquots (1 μL) from a range of MeOH solutions of deoxyluteoskyrin (**6**) (2 mM to 3.9 μM) and rugulosin (**7**) (12 mM to 93 μM), and MeCN solutions of shornephine A (**9**) (0.2 μM to 0.001 μM) and neoasterriquinone (**13**) (27 μM to 12 μM), were analysed by UPLC-DAD (Section 3.6) to generate linear regression calibration curves. These curves were used to quantify metabolite production during time course studies of LPS stimulation trials (Sections 2.9.3 and 2.10.3).

### 2.12. Micro-bioreactor cultivation and LPS stimulation of *Aspergillus niger* (ACM-4993F), *Rhizopus oryzae* (ACM-165F) and *Thanatephorus cucumeris* (ACM-194F)

*Aspergillus niger* (ACM-4993F) was sourced in 1994 as type strain (ATCC 10577), *Rhizopus oryzae* (ACM-165F) was obtained in 1985 from a soy sauce fermentation from Kuala Lumpur, Malaysia, and *Thanatephorus cucumeris* (ACM-194F) was sourced in 1987 as a type strain (ATCC 13289). Micro-bioreactor plates loaded with ISP-2 broth and inoculated with either *A. niger* (ACM-4993F), *R. oryzae* (ACM-165F) or *T. cucumeris* (ACM-194F), were cultivated in the absence and presence of LPS for 10 days, and the resulting analytes were analysed by UPLC-DAD and HPLC-DAD-ESIMS.

### 2.13. Light and fluorescence microscopy analysis of LPS activation of fungal nitric oxide

The NO production was detected using a NO detection kit according to the manufacturer’s instructions, with some modifications. Briefly, four fungal strains were chosen for the detection of NO production, including the LPS-responsive *Penicillium* sp. (ACM-4616F) and *Penicillium* sp. (CMB-TF0411), and the LPS non-responsive *A. brasiliensis* (ACM-4711) and *A. oryzae* (ACM-4669). Inocula were prepared as above (Section 2.3) and the strains were cultivated in the micro-bioreactor for 7 days at 190 rpm, 27°C in ISP-2 broth. To detect the production of NO, fungal cells were washed twice with 10× wash buffer composed of normal saline neutral (pH 7.3) phosphate-buffered saline, after which they were centrifuged at 14,000 rpm for 1 min and the cells transferred to coverglass (12 mm) bottom plates. The NO detection reagent was diluted according to the manufacturer’s instructions by adding the reagent (2.5 μL) to ISP-2 broth (1 mL), after which 100 μL was added to the fungal cells. Following the addition of the NO detection reagent, the plates were incubated for 2 hours at 27°C, after which LPS (15 μL, 0.6 ng/mL) was added and the plates were incubated at 27°C for a further 30 min. In addition to treating cells with LPS, duplicate sets of two forms of negative control were prepared where cells were (i) not treated with LPS or (ii) treated with LPS (15 μL, 0.6 ng/mL) but were subsequently treated with the NO scavenger c-PTIO (40 μM, 100 μL) and incubated for a further 15 min. The fluorescent products from the NO detection kit were measured by confocal microscopy and the images analysed and processed using ImageJ-145 software and NIS-Elements AR version 3.2 64 bit edition.

## 3. Results and discussion

### 3.1. Micro-bioreactor cultivation

**Figure 1.**
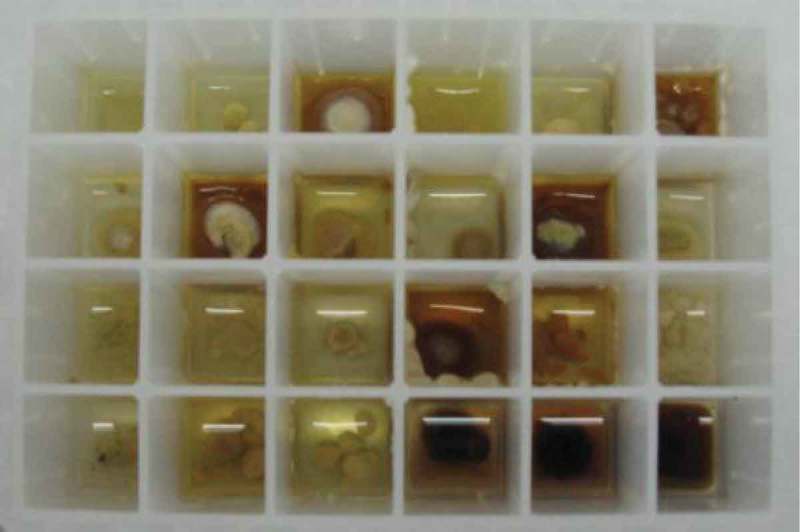
Representative micro-bioreactor cultivation plate.

Discovery scale fermentations often employ liquid shake flask culture to produce secondary metabolite screening samples. When applied to a large numbers of isolates across a range of culture conditions, this process can be time consuming and labour intensive, demanding of laboratory space and resources and restrictive of experimental design. Because our LPS stimulation studies required >1000 individual cultures, we elected to adapt micro-bioreactor (Figure 1) and UPLC-DAD technology to achieve a HTP and high-sensitivity, micro-cultivation and micro-analysis capability (Sections 2.4 and 2.6). As has been shown previously with fungi (Bills et al. 2008), the chemical composition, yields and rates of production of secondary metabolites in micro-bioreactor cultures (1.5 mL) were comparable to those encountered in traditional shake flasks (250 mL) and were highly reproducible and uncompromised by cross-well contamination. We also demonstrated that *in situ* EtOAc extraction yielded analytes ideally suited to UPLC-DAD analysis, generating very rapid (<2 min), high-quality chromatograms (Section 3.6). These analytes were also well suited to longer (>20 min) HPLC-DAD-ESIMS analyses (Section 2.7).

### 3.2. LPS stimulation of fungal secondary metabolism

To test the hypothesis that LPS could stimulate fungal secondary metabolism, the set of 40 fungal strains was challenged with varying concentrations of LPS (100–0.01 ng/mL). Significant changes in the secondary metabolite profile were detected in 6 of the 40 fungi (15%) tested. LPS-responsive fungi included *Penicillium* sp. (ACM-4616), *Penicillium* sp. (CMB-TF0411), *Aspergillus* sp. (CMB-M81F), *A. niger* (ACM-4993F), *R. oryzae* (ACM-165F) and *T. cucumeris* (ACM-194F). Interestingly, LPS stimulation was only observed over a very narrow concentration range (0.3−3 ng/mL) and was suppressed at concentrations >3 ng/mL, suggestive of the involvement of an extracellular receptor akin to the Toll-like receptor (TLR4) that mediates the LPS inflammatory cascade in mammalian cells. Based on these results, we adopted an optimal LPS concentration of 0.6 ng/mL for all subsequent analytical and preparative scale cultures.

### 3.3. *Penicillium* sp. (ACM-4616)

**Figure 2.**
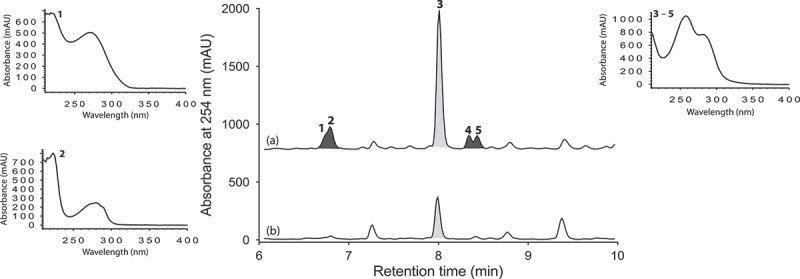
Expansions from the HPLC-DAD-ESIMS chromatograms (254 nm) of EtOAc extracts derived from *Penicillium* sp. (ACM-4616) cultured for 7 days in SDB broth (a) with and (b) without LPS (0.6 ng/mL). Enhanced and activated metabolites are shown in light and dark grey, respectively.

**Figure 3.**
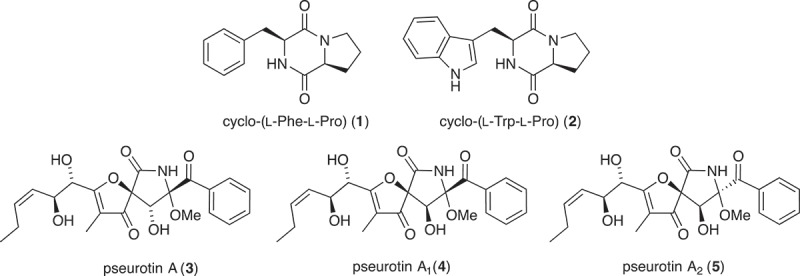
Metabolites **1**−**5** produced by LPS-stimulated *Penicillium* sp. (ACM-4616).

UPLC-DAD analysis of LPS-treated micro-bioreactor cultures of *Penicillium* sp. (ACM-4616) revealed changes to the secondary metabolite profile. HPLC-DAD-ESIMS analyses (Figure 2) of a preparative scale cultivation followed by isolation and characterization confirmed a 289% enhancement in production of the polyketide synthase-non ribosomal peptide synthase hybrid metabolite pseurotin A (**3**), as well as an concurrent activation of pseurotin A_1_ (**4**) and pseurotin A_2_ (**5**) and the diketopiperazines *cyclo*-(L-Phe-L-Pro) (**1**) and *cyclo*-(L-Trp-L-Pro) (**2**) (Figure 3). In this context, the term ‘enhancement’ refers to an increase in production relative to control cultures, whereas ‘activation’ refers to the production of metabolites not observed in control cultures. Pseurotin A (**3**) is a well-known fungal metabolite from the fungus *Pseudeurotium ovalis* (Bloch et al. 1976), whereas pseurotins A_1_ (**4**) and A_2_ (**5**) were only recently reported from a holothurian-derived *Aspergillus fumigatus* (WFZ-25) (Wang et al. 2011). Significantly, recent reports describe the induction of ‘silent’ pseurotin secondary metabolism in *A. fumigatus*, including an account of co-cultivation with a desert soil *Streptomyces bullii* (Rateb et al. 2013) and an account on the impact of hypoxic cultivation (Vödisch et al. 2011). The latter report is noteworthy insofar as LPS is known to stimulate NO production in mammalian cells, which in turn inhibits cellular respiration, leading to metabolic hypoxia (Radi et al. 2002). These observations suggested a link between hypoxic and LPS-mediated stimulation of secondary metabolism that merits future investigation.

### 3.4. *Penicillium* sp. (CMB-TF0411)

**Figure 4.**
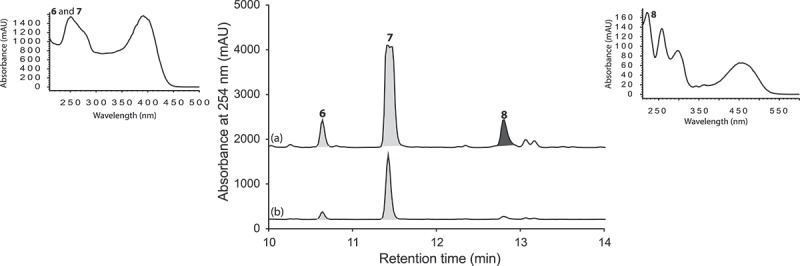
Expansions from the HPLC-DAD-ESIMS chromatograms (254 nm) of EtOAc extracts derived from *Penicillium* sp. (CMB-TF0411) cultured for 10 days in ISP-2 broth (a) with and (b) without LPS (0.6 ng/mL). Enhanced and activated metabolites are shown in light and dark grey, respectively.

**Figure 5.**
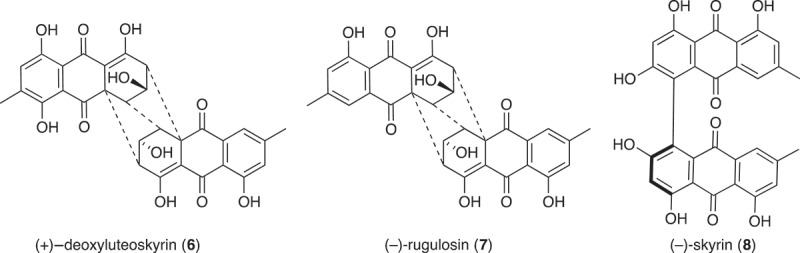
Metabolites produced by LPS-stimulated *Penicillium* sp. (CMB-TF0411).

UPLC-DAD analysis of LPS-treated micro-bioreactor cultures of *Penicillium* sp. (CMB-TF0411) revealed changes to the secondary metabolite profile. HPLC-DAD-ESIMS analyses (Figure 4) and preparative scale cultures confirmed 736 and 101% enhancements in (+)-deoxyluteoskyrin (**6**) ([*α*]_D_ + 85) and (–)-rugluosin (**7**) ([*α*]_D_ – 22), respectively, and activation of (−)-skyrin (**8**) ([*α*]_D_ – 16) (Figure 5). While (–)-rugluosin (**7**) has been reported from the treatment of (–) flavoskyrin with pyridine (Seo et al. 1973), to the best of our knowledge (+)-deoxyluteoskyrin (**6**) and (−)-skyrin (**8**) have not been previously described. By comparison, the enantiomeric (+)-rugulosin first reported from *Penicillium rugulosum* (Breen et al. 1955) and subsequently revised (Takeda et al. 1973) is known to co-occur in *Pyrobaculum islandicum* with the enantiomers of **6** and **8** (Antonowitz et al. 1994). To explore the effect of culture age on the stimulatory effect of LPS on *Penicillium* sp. (CMB-TF0411), we monitored the levels of **6** and **7** during a 20-day fermentation and observed that while LPS enhanced the production of **6** and **7** from days 5 to 10, by day 20, the secondary metabolite profile was independent of LPS treatment and was dominated by **7**. These observations emphasized the need for cautious interpretation of the impact of LPS on secondary metabolism and reinforced the need to assess potential stimulatory effects across multiple time points. In the case of *Penicillium* sp. (CMB-TF0411), we concluded that although LPS influenced the production of (–)-rugulosin biosynthetic intermediates at an early phase in the cultivation, ultimately the chemical composition of the mature culture was not significantly changed.

### 3.5. *Aspergillus* sp. (CMB-M81F)

**Figure 6.**
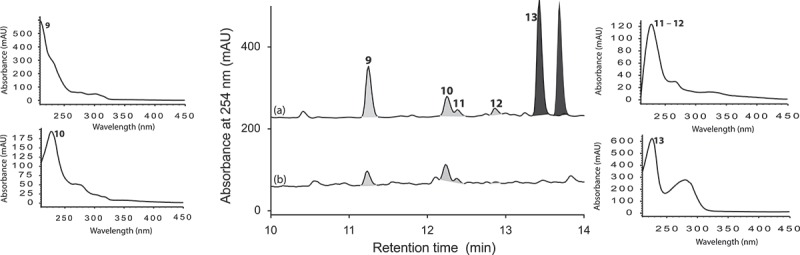
Expansions from the HPLC-DAD-ESIMS chromatograms (254 nm) of EtOAc extracts derived from *Aspergillus* sp. (CMB-M81F) cultured for 120 days in M1 broth with 3.3% artificial ocean sea salt (a) with and (b) without LPS (0.6 ng/mL). Enhanced and activated metabolites are shown in light and dark grey, respectively.

**Figure 7.**
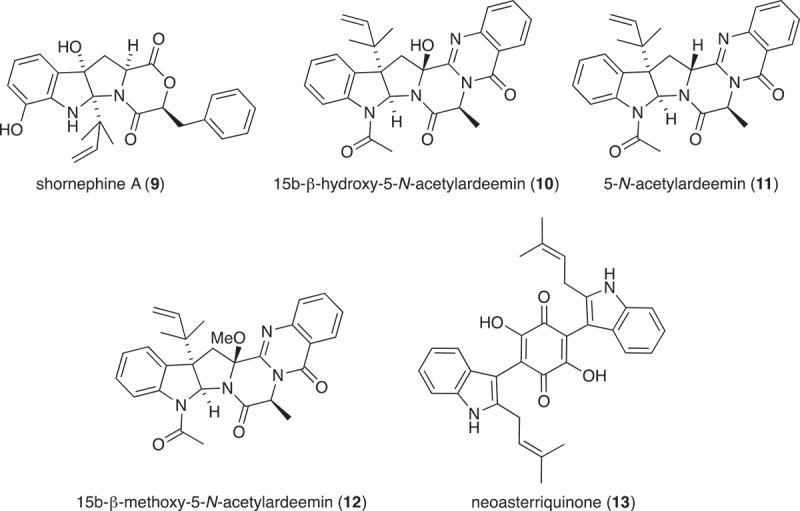
Metabolites produced by LPS-activated *Aspergillus* sp. (CMB-M81F).

*Aspergillus* sp. (CMB-M81F) was a particularly challenging strain that required exceptionally long cultivation times (>120 days) to produce a meaningful secondary metabolite profile. We were, therefore, encouraged to observe that it was sensitive to LPS treatment and were hopeful that secondary metabolite production might be accelerated. UPLC-DAD analysis of 120 day LPS-treated micro-bioreactor cultures of *Aspergillus* sp. (CMB-M81F) revealed significant changes to the secondary metabolite profile. HPLC-DAD-ESIMS analyses (Figure 6) of the preparative scale (120-day) culture confirmed enhanced production of shornephine A (**9**) (404%), 15b-β-hydroxy-5-*N*-acetylardeemin (**10**) (277%), 5-*N*-acetylardeemin (**11**) (300%) and 15b-β-methoxy-5-*N*-acetylardeemin (**12**) (208%) and activation of the production of neoasterriquinone (**13**) (Figure 7). While the natural products **9** and **12** have yet to be reported in the primary scientific literature (manuscript in preparation), ardeemins **10** and **11** were first reported from *Aspergillus fischeri* var. *brasiliensis* (Hochlowski et al. 1993) and **13** from *A. terreus* (Kaji et al. 1994). To explore whether LPS could accelerate secondary metabolite production (and thereby reduce cultivation times), we monitored LPS-treated and control micro-bioreactor cultures to quantify the levels of **9** and **13** over a 160-day period. In control cultures, **9** first appeared at day 70, with maximum production at day 160, whereas the addition of LPS accelerated these milestones to day 40 and day 120, respectively. Not only did LPS substantially accelerate the production of **9**, but also its yield increased 600%. Similarly, in LPS-treated cultures of *Aspergillus* sp. (CMB-M81F), the production of **13** was activated by day 40, with maximum production achieved at day 80 and maintained until day 160.

### 3.6. Additional LPS stimulation events

**Figure 8.**
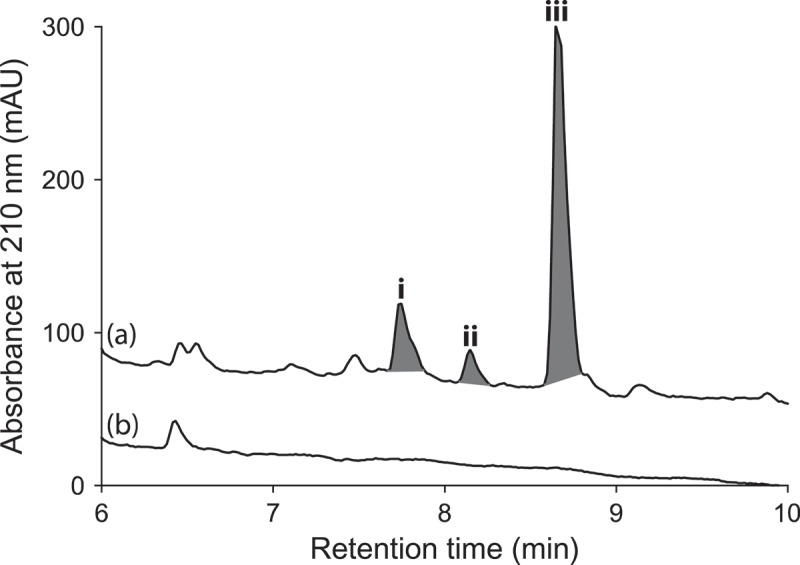
Expansions from the HPLC-DAD-ESIMS chromatograms (254 nm) of EtOAc extracts derived from *Aspergillus niger* (ACM-4993F) cultured for 10 days in ISP-2 broth (a) with and (b) without LPS (0.6 ng/mL). Activated metabolites are shown in dark grey.

**Figure 9.**
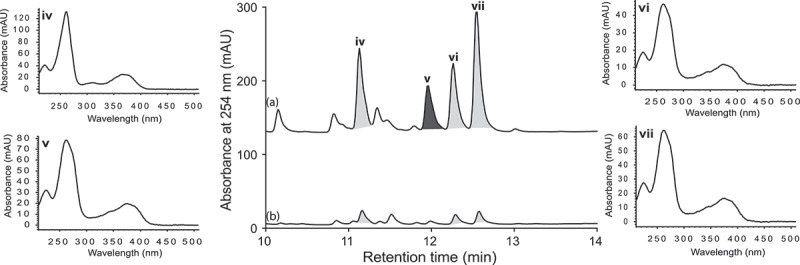
Expansions from the HPLC-DAD-ESIMS chromatograms (254 nm) of EtOAc extracts derived from *Rhizopus oryzae* (ACM-165F) cultured for 10 days in ISP-2 broth (a) with and (b) without LPS (0.6 ng/mL). Enhanced and activated metabolites are shown in light and dark grey, respectively.

**Figure 10.**
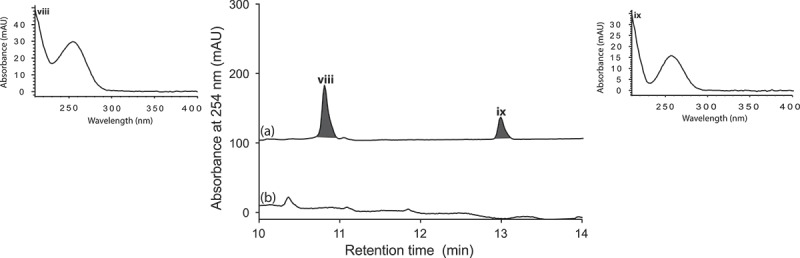
Expansions from the HPLC-DAD-ESIMS chromatograms (254 nm) of EtOAc extracts derived from *Thanatephorus cucumeris* (ACM-194F) cultured for 10 days in ISP-2 broth (a) with and (b) without LPS (0.6 ng/mL). Activated metabolites are shown in dark grey.

UPLC-DAD analysis of LPS-treated micro-bioreactor cultures of *A. niger* (ACM-4993F), *R. oryzae* (ACM-165F) and *T. cucumeris* (ACM-194F) revealed significant changes to secondary metabolite profiles. Even though *A. niger* is a prolific producer of secondary metabolites, HPLC-DAD-ESIMS analyses of LPS-stimulated *A. niger* (ACM-4993F) (Figure 8) cultures confirmed the activation of (**i–iii**). Likewise, HPLC-DAD-ESIMS analyses of LPS-stimulated *R. oryzae* (ACM-165F) (Figure 9) cultures confirmed 354, 471 and 649% enhancement of (**iv–vi**) and activation of (**vii**), while LPS-stimulated *T. cucumeris* (ACM-194F) (Figure 10) cultures revealed activation of (**viii**) and (**ix**). Activation of *R. oryzae* and *T. cucumeris* are particularly noteworthy as neither are recognized in the scientific literature for the production of secondary metabolites, with genomic analyses suggesting that *R. oryzae* (Ma et al. 2009) and *T. cucumeris* (Wibberg et al. 2013) have limited capacity to produce secondary metabolites.

**Figure 11.**
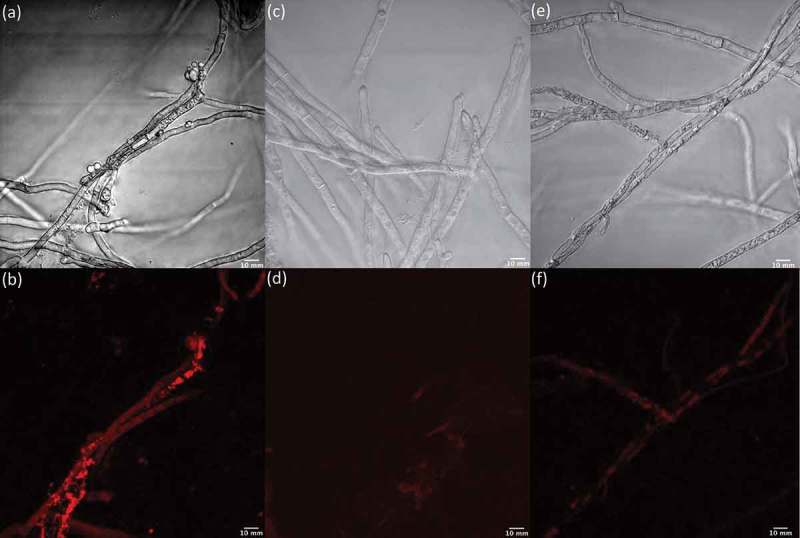
Light and fluorescence microscopy images of *Penicillium* sp. (ACM-4616) cells exposed to an NO detection kit in the presence (a/b) and absence (e/f) of LPS and in the presence of LPS and an NO quenching agent (c/d).

**Figure 12.**
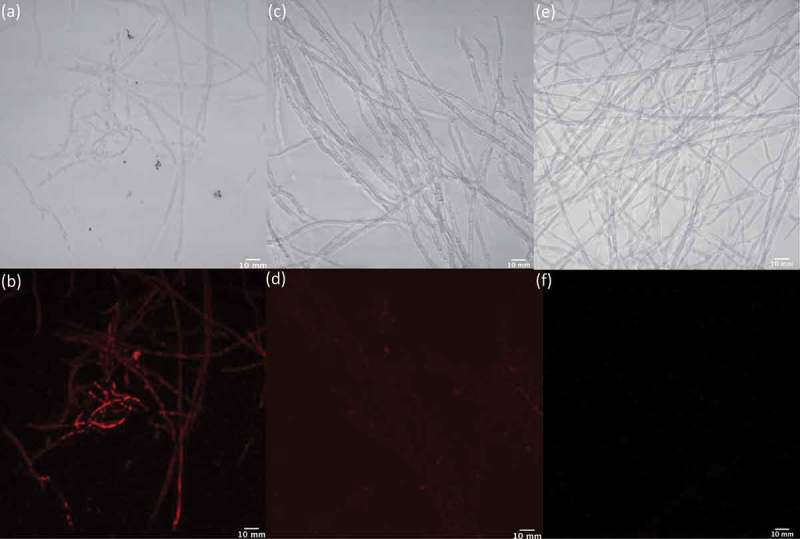
Light and fluorescence microscopy images of *Penicillium* sp. (CMB-TF0411) cells exposed to an NO detection kit in the presence (a/b) and absence (e/f) of LPS and in the presence of LPS and an NO quenching agent (c/d).

To determine whether the treatment of fungal cells with LPS correlated with an increase in NO production, as is well documented in mammalian cells, we used an NO detection kit together with light and fluorescence microscopy to image *Penicillium* sp. (ACM-4616) (Figure 11) and *Penicillium* sp. (CMB-TF0411) (Figure 12) in the presence and absence of LPS and in the presence of LPS plus the NO scavenger c-PTIO. Although the NO detection kit and c-PTIO are better known as tools for fluorescent imaging of NO levels in mammalian cells, we determined that they could be used to image NO levels in fungal and bacterial cells (data not shown). Both *Penicillium* sp. (ACM-4616) and *Penicillium* sp. (CMB-TF0411) exhibited strong fluorescence when treated with LPS (Figures 11b and 12b), consistent with LPS-mediated activation of NO production. Importantly, neither *Penicillium* spp. exhibited fluorescence in the absence of LPS nor in the presence of LPS and the NO quenching agent c-PTIO. By comparison, two control fungi that did not exhibit LPS-stimulated changes to their secondary metabolism, *A. brasiliensis* (ACM-4711) and *A. oryzae* (ACM-4669), revealed negligible levels of NO (fluorescence) when challenged with LPS. Albeit preliminary, these observations nevertheless suggest that LPS stimulation of fungal secondary metabolism may be correlated with LPS activation of fungal NO production – consistent with our hypothesis that LPS stimulates a pseudo-inflammatory response in selected fungi. Further investigation will be needed to understand the mechanism of these responses at a transcriptional level.

## 4. Conclusion

The observations outlined above demonstrate that the addition of LPS to fungal cultures can modulate secondary metabolite profiles in some fungi. Implementation of an integrated HTP micro-cultivation and micro-analysis methodology enabled the cost-effective and timely analysis of a matrix of >1000 cultures and determined that six (15%) of the 40 fungi tested responded to LPS with changes to secondary metabolite profiles. Furthermore, we established an optimal LPS concentration (0.6 ng/mL) and confirmed that stimulatory responses observed in micro-bioreactor cultivations (1.5 mL) could be scaled up (>400 mL). Detailed examination of the LPS stimulation of three fungi revealed three distinctive stimulatory responses, characterized by enhancement, activation and acceleration of secondary metabolism expression. More specifically, LPS treatment of *Penicillium* sp. (ACM-4616) enhanced pseurotin A (**3**), and activated pseurotin A_1_ (**4**) and pseurotin A_2_ (**5**) biosynthesis, whereas LPS treatment of *Penicillium* sp. (CMB-TF0411) altered anthraquinone biosynthesis in early-phase cultivations, but did not ultimately have a significant impact on the chemical composition of mature cultures. By contrast, LPS treatment of *Aspergillus* sp. (CMB-M81F) substantially accelerated and enhanced the biosynthesis of shornephine A (**9**) and ardeemins **10–13** and activated production of neoasterriquinone (**13**). Fluorescence microscopy and assays with NO-sensitive fluorescent dyes and quenching agents provided evidence that LPS stimulation of secondary metabolism may be correlated with activation of NO production, suggestive of a chemical defensive response. Illustrative of the potential of LPS as a tool for influencing secondary metabolism, cultures of *A. niger* (ACM-4993F), *R. oryzae* (ACM-165F) and *T. cucumeris* (ACM-194F) were sensitive to LPS stimulation. These results support the hypothesis that LPS stimulation can be a valuable tool to expand the molecular discovery potential of fungal strains.
